# Contribution of prepregnancy body mass index and gestational weight gain to caesarean birth in Canada

**DOI:** 10.1186/1471-2393-14-106

**Published:** 2014-03-18

**Authors:** Susie Dzakpasu, John Fahey, Russell S Kirby, Suzanne C Tough, Beverley Chalmers, Maureen I Heaman, Sharon Bartholomew, Anne Biringer, Elizabeth K Darling, Lily S Lee, Sarah D McDonald

**Affiliations:** 1 Maternal and Infant Health Section, Surveillance and Analysis Division, Public Health Agency of Canada, 785 Carling Avenue, 6804A 4th Floor, Ottawa, ON K1A 0K9, Canada; 2 Reproductive Care Program of Nova Scotia, Halifax Professional Centre, Suite 700, 5991 Spring Garden Road, Halifax, NS B3H 1Y6, Canada; 3 Department of Community and Family Health, College of Public Health, University of South Florida, Bruce B. Downs Blvd. MDC 56, Tampa, FL 13201, USA; 4 Departments of Paediatrics and Community Health Sciences, Faculty of Medicine, University of Calgary, 2500 University Dr. NW, Calgary, AB T2N 1N4, Canada; 5 Department of Obstetrics and Gynaecology, Ottawa Hospital Research Institute, University of Ottawa, 501 Smyth Road, Ottawa, ON K1H 8L6, Canada; 6 Faculty of Nursing, Helen Glass Centre for Nursing, University of Manitoba, 89 Curry Place, Winnipeg, MB R3T 2N2, Canada; 7 Department of Family and Community Medicine, Mount Sinai Hospital, University of Toronto, Queen’s Park, Toronto, ON M5S 2C5, Canada; 8 Midwifery Education Program, Laurentian University, 935 Ramsey Lake Road, Sudbury, ON P3E 2C6, Canada; 9 Perinatal Services British Columbia, Provincial Health Services Authority, 3rd Floor West Tower, 555 West 12th Ave, Vancouver, BC V5Z 3X7, Canada; 10 Departments of Obstetrics & Gynecology, Radiology, and Clinical Epidemiology & Biostatistics, McMaster University, 1280 Main Street West, Hamilton, ON L8S 4L8, Canada

**Keywords:** Population attributable fractions, Maternal weight, Caesarean section

## Abstract

**Background:**

Overweight and obese women are known to be at increased risk of caesarean birth. This study estimates the contribution of prepregnancy body mass index (BMI) and gestational weight gain (GWG) to caesarean births in Canada.

**Methods:**

We analyzed data from women in the Canadian Maternity Experiences Survey who had a singleton term live birth in 2005-2006. Adjusted odds ratios for caesarean birth across BMI and GWG groups were derived, separately for nulliparous women and parous women with and without a prior caesarean. Population attributable fractions of caesarean births associated with above normal BMI and excess GWG were calculated.

**Results:**

The overall caesarean birth rate was 25.7%. Among nulliparous and parous women without a previous caesarean birth, rates in obese women were 45.1% and 9.7% respectively, and rates in women who gained above their recommended GWG were 33.5% and 8.0% respectively. Caesarean birth was more strongly associated with BMI than with GWG. However, due to the high prevalence of excess GWG (48.8%), the proportion of caesareans associated with above normal BMI and excess GWG was similar [10.1% (95% CI: 9.9-10.2) and 10.9% (95% CI: 10.7-11.1) respectively]. Overall, one in five (20.2%, 95% CI: 20.0-20.4) caesarean births was associated with above normal BMI or excess GWG.

**Conclusions:**

Overweight and obese BMI and above recommended GWG are significantly associated with caesarean birth in singleton term pregnancies in Canada. Strategies to reduce caesarean births must include measures to prevent overweight and obese BMI prior to conception and promote recommended weight gain throughout pregnancy.

## Background

The prevalence of overweight and obesity, defined as a body mass index (BMI) of 25-29.9 kg/m^2^ and ≥ 30 kg/m^2^ respectively, has been increasing globally [1]. In Canada, based on measured height and weight, the prevalence of obesity among adult women rose from 16% in 1978 to 23% in 2004 [2]. Correspondingly, rates of obesity are also increasing among pregnant women. Overweight and obese women are known to be at increased risk of serious pregnancy complications including caesarean birth [3-6]. These caesarean births in turn increase women’s risk of infection, haemorrhage, damage to the intestines or bladder, and negatively affect early parenting outcomes [7-9]. Caesarean births also increase the risk of long-term complications such as abnormal placentation during subsequent pregnancies and place excess strain on the healthcare system [7,10,11]. Canadian caesarean birth rates rose from 18% in 1995-1996 to 28% in 2010-2011 [12,13].

The concomitant increase in overweight and obesity and caesarean births make it important to study to what degree maternal weight is contributing to these births. During pregnancy, maternal weight is a product of both prepregnancy body mass index (BMI), hereafter referred to as BMI, and gestational weight gain (GWG). Estimating the magnitude of the independent as well as joint association between these determinants and caesarean births is essential for designing interventions that promote healthy pregnancy outcomes. However, to date, few studies have quantified the proportion of caesarean births at the population level that are associated with above normal BMI [14-16] and no studies have quantified the proportion associated with excess GWG. Data from the Canadian Maternity Experiences Survey provided a unique opportunity to address this issue for Canada.

## Methods

### Study population

This study used data from the Public Health Agency of Canada’s Canadian Maternity Experiences Survey (MES). The MES was a cross-sectional survey of a stratified random sample of women who had a singleton live birth in Canada between November 2005 and May 2006. Women were identified using recent births drawn from a Census-based sampling frame. Women were eligible for the study if they were at least 15 years of age and were living with their infant at the time of data collection. Women living on First Nations reserves or in institutions were excluded. Data were collected by female interviewers between October 2006 and January 2007 using a computer-assisted telephone interview application. The majority (97%) of interviews were conducted between five and nine months postpartum. Out of 8,244 eligible women, 6,421 (78%) agreed to participate. In consideration of the sample design and non-participation, each MES record was assigned a sampling weight. The 6,421 respondents were thus weighted to represent 76,508 women nationally who had a singleton live birth between November 2005 and May 2006. Survey questions covered a broad range of pregnancy, birth and postpartum experiences. All data were based on women’s reports. Detailed information on the survey’s development, methodology and content has been reported elsewhere [17].

We excluded women with missing information on BMI or GWG (n = 79), as these were the principal determinants of interest. Mode of birth information was complete. We also excluded preterm births (< 37 weeks gestation) (n = 568) and women who were less than 18 years old (n = 183), as the BMI classification used was derived for ages 18 and older. These exclusions resulted in a final sample of 5,591 women weighted to represent 67,058 women.

### Outcomes

The primary outcome was caesarean birth (planned and unplanned). Caesarean births were classified as planned if the decision about the mode of birth was made before the woman went into labour.

### Determinants

Prepregnancy BMI and GWG were the principal exposures of interest. They were derived from the following questions:

i) *How tall are you without shoes on?*

ii) *Just before your pregnancy, how much did you weigh?*

iii) *How much weight did you gain during your pregnancy?*

We categorized women according to the World Health Organization (WHO) standard as either being underweight (BMI < 18.5 kg/m^2^), normal weight (18.5 ≤ BMI < 25), overweight (25 ≤ BMI < 30) or obese (BMI ≥ 30). Women were also classified according to the Institute of Medicine’s recommended GWG ranges (Table 1) [18], as having gained above, within or below the recommended weight for their BMI.

**Table 1:** Gestational weight gain (GWG) recommendations

**Prepregnancy BMI (kg/m**^**2**^**)**	**Recommended GWG (kg)**
Underweight: BMI < 18.5	12.5 - 18.0
Normal weight: 18.5 ≤ BMI < 25	11.5 - 16.0
Overweight : 25 ≤ BMI < 30	7.0 - 11.5
Obese: BMI ≥ 30	5.0 - 9.0

### Covariates

We studied additional reproductive, health care, sociodemographic and psychosocial characteristics as potential confounders of the association between BMI, GWG and mode of birth. Birthweight-for-gestational-age was derived using a Canadian reference to categorize infants below the standard 10^th^ percentile as small-for-gestational age (SGA) and those above the standard 90^th^ percentile as large-for-gestational age (LGA) [19]. Sociodemographic variables assessed included the household’s low income cut-off level (LICO), which is a measure of the income threshold below which a family will likely spend 20 percentage points more than the average family on food, shelter and clothing [20]. Ethnicity was based on mother’s country of birth, grouped according to world regions; mothers born in Canada were categorized as Aboriginal off-reserve and non-Aboriginal. The MES questions for variables whose definitions are not self-evident are indicated in Table 2. Categorizations (for non-dichotomized variables) used in analyses are indicated in Table 3 in the results section.

**Table 2:** Definitions of selected covariates

**Variable**	**MES question**
***Reproductive/health care factors***	
Health care provider started labour	*Did your healthcare provider try to start or induce your labour by the use of medication or some other technique?*
Prepregnancy health problems	*Before your pregnancy, did you have any medical conditions or health problems that required you to take medication for more than 2 weeks, have special care or extra tests during your pregnancy?*
During pregnancy health problems	*During your pregnancy, did you develop any new medical conditions or health problems that required you to take medication for more than 2 weeks, have special care or extra tests?*
Antenatal care provider	*From which type of healthcare provider, such as an obstetrician, family doctor or midwife, did you receive most of [your prenatal] care?*
***Psychosocial factors***	
Support	*During your pregnancy, how often was support available to you when you needed it?* None/a little/some/most/all of the time.
Stressful life events	High stress was defined as experiencing 3 or more of the following 12 events in the year before the birth: *close family member hospitalized, move to a new address, homelessness, woman or partner lost job, woman or partner went to jail, more than usual arguments with partner, partner not wanting pregnancy, separation or divorce, bills that could not be paid, a physical fight, someone close having a problem with alcohol or drugs, someone close dying.*
History of depression	*Before your pregnancy, had you ever been prescribed anti-depressants or been diagnosed with depression?*

### Statistical analysis

Percentages were used to report observed distributions of BMI and GWG across maternal characteristics. We calculated adjusted odds ratios (ORs) for having a caesarean birth using multivariable logistic regression. With the exception of maternal age, all variables were treated as categorical in regression models. Records with missing values for covariates other than LICO were excluded from models (< 4%). Due to a larger number of missing LICO values (8.0%), a missing category was included for this variable. We calculated ORs across BMI and GWG groups for caesarean births overall as well as for unplanned and planned caesarean births. Normal BMI and within recommended GWG were the reference groups.

BMI and GWG were included in all multivariable models in order to estimate their independent associations (ORs) with caesarean birth. Other covariates were selected into models purposefully using the following steps [21]. Based on the Wald test from univariable logistic regression models, we initially included any variable with a p-value below 0.25. Covariates were then removed from the model if they were statistically non-significant and not a confounder. Significance was evaluated at the 0.05 level and confounding as a change of 15% or higher in the effect of BMI or GWG on the mode of birth outcome being modeled. To address significant interaction between parity, prior caesarean, BMI, GWG and mode of birth, we stratified our analysis into three subgroups: nulliparous, parous without previous caesarean and parous with previous caesarean. As health problems before or during pregnancy and a health care provider trying to induce labour may be on the causal pathway between BMI, GWG and mode of birth, we assessed results from models with and without these variables.

The contribution to caesarean births of overweight or obese BMI and more than recommended GWG was estimated using population attributable fractions (PAFs). The calculation of PAFs has the advantage of incorporating the increased risk due to high BMI or GWG and the prevalence of these two determinants, in order to provide an estimate of the potential reduction in caesarean birth if high BMI and GWG were eliminated. We calculated PAFs directly from our multivariable logistic regression models using the sequential and average attributable fraction method which takes into account that ORs are adjusted for confounders [22].

All analyses were carried out using sampling weights. We calculated variances using bootstrap weights to capture the variability introduced by the sample design and weighting adjustments [23]. We used SAS EG software, Version 5.1, copyright SAS Institute Inc. [24]. Review by an ethics board was not required, as the MES data are anonymous and this study did not generate identifying information.

## Results

The prevalence of overweight and obese BMI was 20.9% and 13.3% respectively. Almost one-half (48.8%) of women gained above the recommended weight for their BMI. The two determinants were strongly associated with each other; 60.3% of obese versus 30.2% of underweight women gained more than the recommended weight and 12.2% of obese versus 24.8% of underweight women gained less than the recommended weight. The distribution of covariates within BMI and GWG groups is shown in Table 3.

**Table 3:** Distribution (%) of covariates across prepregnancy body mass index (BMI) and gestational weight gain (GWG) categories*

	**Prepregnancy BMI**				**Recommended GWG**		
	**Under-weight**	**Normal weight**	**Over-weight**	**Obese**	**Below**	**Within**	**Above**
Percent of study sample	5.9	60.0	20.9	13.3	18.1	33.1	48.8
***Reproductive/health care factors***							
Maternal age at birth^**^							
≤24	27.3	14.7	13.5	14.3	14.5	11.9	17.5
25-29	29.4	33.8	34.8	38.0	32.0	33.8	35.5
30-34	30.2	33.3	34.3	32.8	32.3	35.9	31.8
≥35	13.2	18.2	17.4	15.0	21.2	18.3	15.2
Nulliparous	50.3	47.5	37.8	40.6	39.6	41.2	49.0
Birthweight-for-gestational age^†^							
SGA	17.0	8.2	7.4	6.2	13.6	8.6	6.1
AGA	78.2	85.2	78.4	77.0	81.0	81.6	79.5
LGA	4.7	9.6	14.2	16.8	5.4	9.8	14.4
Health care provider started labour^‡^	36.6	42.5	47.6	58.9	41.1	41.6	49.3
Prepregnancy health problems	14.1	12.7	16.7	19.3	15.2	14.3	14.3
During pregnancy health problems	17.3	22.3	23.2	32.7	27.1	22.2	23.0
Antenatal care provider							
Obstetrician/ gynaecologist	59.0	58.6	56.6	60.2	61.1	58.9	57.1
General practitioner	36.0	34.2	36.5	35.7	33.1	34.4	36.0
Midwife/nurse	5.0	7.3	6.9	4.2	5.8	6.7	6.9
***Sociodemographic factors***							
Low-income-cut-off							
≤LICO	29.7	15.8	18.5	21.0	19.8	16.3	18.2
>LICO	57.6	76.3	73.6	72.3	72.5	75.5	73.8
Missing	12.7	7.9	7.9	6.7	7.7	8.2	8.0
Education							
Less than high school	14.4	5.5	6.6	8.2	6.5	4.5	8.1
High school graduate	21.7	17.5	20.6	24.6	18.3	19.0	20.0
Post-secondary diploma	31.8	36.2	39.3	43.2	37.6	35.7	38.8
University graduate	32.1	40.8	33.4	24.0	37.6	40.9	33.2
Region/province							
Atlantic	3.4	4.8	7.2	9.2	3.9	5.4	6.7
Quebec	25.6	24.8	25.3	20.5	24.4	26.2	23.1
Ontario	40.7	38.7	34.5	40.3	39.1	37.8	38.0
Prairies	17.6	18.0	22.0	20.3	18.6	18.0	20.0
British Columbia	12.6	13.4	10.5	9.12	13.5	12.1	11.7
Territories	0.1	0.4	0.5	0.6	0.6	0.4	0.4
Urban residence	87.2	82.5	80.4	79.6	84.9	82.3	80.7
Ethnicity (country of birth)							
Canada/Aboriginal off-reserve	2.4	3.4	4.4	6.1	2.5	2.7	5.3
Canada/non-Aboriginal	57.2	71.0	77.7	78.8	65.5	72.5	75.4
Europe/Western	4.7	7.1	4.0	4.8	6.3	5.9	5.9
Africa/Mid East/Latin	10.6	7.3	7.7	5.1	10.2	7.9	5.8
East/South Asia/Pacific	25.2	11.2	6.2	5.4	15.5	11.1	7.6
Married^††^	84.2	93.1	93.0	89.2	92.3	93.9	90.6
***Psychosocial factors***							
No/some social support	12.6	12.3	12.8	14.5	14.0	12.6	12.3
3+ stressful events	22.3	15.2	16.3	21.4	15.0	15.3	18.2
History of depression	14.6	13.4	16.4	22.5	15.0	13.7	16.5
Smoked 3^rd^ trimester	15.0	9.1	10.2	12.2	10.4	8.2	11.2
Drank alcohol in pregnancy	8.2	12.4	10.1	8.0	10.4	12.4	10.5

^*^Some columns do not sum to 100% due to rounding. ^**^Regression models used continuous age variable. ^†^SGA: small-for-gestational-age, AGA: average-for-gestational-age, LGA: large-for-gestational-age. ^‡^Among women who attempted vaginal birth. ^††^Married or common law.

### Association between caesarean birth and prepregnancy BMI and GWG

The overall caesarean birth rate was 25.7%, with substantial variation across parity and prior caesarean group strata. Among nulliparous women, parous women with no prior caesarean and parous women with a prior caesarean, caesarean birth rates were 29.6%, 5.8% and 80.2% respectively. Among nulliparous and parous women without a previous caesarean, rates in obese women were 45.1% and 9.7% respectively, and rates in women who gained above their recommended GWG were 33.5% and 8.0% respectively (Table 4). The incidence of caesarean births increased in all groups as BMI and GWG increased, except for GWG among parous women with a prior caesarean. In this group the trend was reversed; but adjusted ORs were not significantly different (Table 4). The high caesarean birth rates (above 75%) in parous women with a prior caesarean and low rates (below 10%) in parous women with no prior caesarean limited the scope for detecting significant decreases or increases in risk in these groups.

**Table 4:** Crude risks (%) and adjusted* odds ratios (ORs) for caesarean birth, by parity and previous caesarean status

	**Caesarean birth**		**Unplanned caesarean**		**Planned caesarean**	
	**%**	**OR (95% CI)**	**%**	**OR (95% CI)**	**%**	**OR (95% CI)**
**All women**						
Underweight	20.9	1.01 (0.74,1.38)	8.3	0.82 (0.52,1.29)	12.6	1.25 (0.82,1.91)
Normal weight	22.9	1	11.0	1	11.9	1
Overweight	28.3	**1.23 (1.04,1.47)**	13.5	**1.38 (1.08,1.76)**	14.8	1.10 (0.88,1.37)
Obese	36.8	**1.95 (1.61,2.36)**	19.1	**2.29 (1.77,2.96)**	17.6	**1.45 (1.13,1.85)**
GWG < recommended	20.1	0.89 (0.71,1.10)	7.6	0.77 (0.56,1.06)	12.4	0.99 (0.75,1.30)
GWG = recommended	22.8	1	10.3	1	12.6	1
GWG > recommended	29.8	**1.36 (1.17,1.59)**	15.7	**1.40 (1.12,1.74)**	14.1	**1.23 (1.01,1.51)**
**Nulliparous**						
Underweight	22.3	0.96 (0.62,1.50)	12.9	0.76 (0.45,1.27)	9.4	1.53 (0.70,3.34)
Normal weight	25.9	1	19.7	1	6.2	1
Overweight	34.9	**1.37 (1.05,1.78)**	27.4	**1.36 (1.02,1.81)**	7.5	1.18 (0.75,1.85)
Obese	45.1	**2.29 (1.72,3.06)**	37.7	**2.41 (1.78,3.25)**	7.3	1.13 (0.63,2.03)
GWG < recommended	23.6	0.87 (0.64,1.19)	15.4	0.79 (0.54,1.15)	7.9	1.08 (0.63,1.84)
GWG = recommended	25.9	1	19.5	1	6.4	1
GWG > recommended	33.5	**1.35 (1.08,1.70)**	26.9	**1.43 (1.12,1.84)**	6.6	0.98 (0.66,1.48)
**Parous, no prior caesarean**						
Underweight	3.5	0.84 (0.22,3.17)	2.6	1.41 (0.29,6.89)	0.9	0.37 (0.01,13.96)
Normal weight	4.8	1	2.1	1	2.7	1
Overweight	6.6	1.01 (0.61,1.69)	4.2	1.38 (0.71,2.71)	2.4	0.66 (0.27,1.61)
Obese	9.7	**2.03 (1.17,3.53)**	5.3	**2.26 (1.04,4.87)**	4.4	1.73 (0.78,3.83)
GWG < recommended	3.4	0.79 (0.40,1.57)	1.0	0.45 (0.13,1.56)	2.4	1.20 (0.48,3.02)
GWG = recommended	4.5	1	2.5	1	2.0	1
GWG > recommended	8.0	**1.75 (1.13,2.71)**	4.4	1.75 (0.96,3.20)	3.5	1.68 (0.85,3.32)
**Parous, prior caesarean**						
Underweight	77.3	1.37 (0.43,4.36)	6.9	1.13 (0.06,20.79)	70.4	1.26 (0.43,3.65)
Normal weight	77.9	1	5.9	1	72.1	1
Overweight	84.3	1.53 (0.85,2.77)	8.0	1.67 (0.61,4.62)	76.3	1.18 (0.69,2.01)
Obese	82.3	1.50 (0.77,2.91)	9.7	2.01 (0.71,5.66)	72.7	1.08 (0.62,1.91)
GWG < recommended	82.2	1.14 (0.57,2.29)	8.5	1.22 (0.39,3.79)	73.7	1.05 (0.55,2.00)
GWG = recommended	80.0	1	7.6	1	72.4	1
GWG > recommended	79.8	1.03 (0.62,1.71)	6.4	0.76 (0.29,1.96)	73.4	1.14 (0.71,1.81)

[Statistically significant values are bolded.]

*All women: BMI models adjusted for GWG and GWG models adjusted for BMI; also adjusted for maternal age, parity, weight-for-gestational age, prepregnancy health problems, antenatal care provider, low-income-cut-off, educational attainment, province of residence, ethnicity, support, stress and history of depression. Parity/previous caesarean subgroups: adjusted for same covariates, except parity.

The adjusted risk of caesarean birth did not differ significantly between underweight and normal-weight women; it also did not differ significantly between women who gained less than the recommended amount and those who gained within the recommended amount (Table 4). The risk was significantly elevated among women who were overweight (OR = 1.23 [1.04-1.47]), obese (OR = 1.95 [1.61-2.36]), or had gained more than the recommended amount (OR = 1.36 [1.17-1.59]). Among nulliparous women who were overweight, obese or above their recommended GWG, the overall risk of caesarean birth was increased, with most of the increase attributable to unplanned caesareans. Among parous women with no prior caesarean, the risk of caesarean birth was low, but significantly elevated among those who were obese (OR = 2.03 [1.17-3.53]) or above their recommended GWG (OR = 1.75 [1.13-2.71]). There was no significant relationship between caesarean births and BMI or GWG among women with a previous caesarean (Table 4). Adjusting for women’s reports that their health care provider tried to induce labour, or that they experienced health problems before or during pregnancy, did not significantly change these risk patterns (data not shown).

### Population attributable fractions of caesarean births associated with BMI and GWG

The fractions of caesarean births associated with overweight or obese BMI and more than recommended GWG are presented in Table 5. Among all women, 10.1% (9.9-10.2) of caesareans were associated with overweight or obese BMI and 10.9% (10.7-11.1) were associated with above recommended GWG. One in five caesareans (20.2% [20.0-20.4]) was associated with either above normal BMI or excess GWG. Results were similar for nulliparous women. In parous women with no previous caesarean, the proportion of caesareans associated with above recommended GWG was twice that of overweight or obese BMI (23.6% [23.0-24.2] versus 10.9% [10.4-11.4]). Almost one third (31.8% [31.3-32.4]) of caesarean births in this group were associated with either above normal BMI or excess GWG.

**Table 5:** Adjusted* population attributable fractions (PAFs) of caesarean births associated with overweight or obese prepregnancy body mass index (BMI) or above recommended gestational weight gain (GWG)

	**PAF (%, 95% confidence interval)**			
	**All women**	**Nulliparous**	**Parous, no prior caesarean**	**Parous, prior caesarean**
Overweight or obese (BMI ≥ 25)	10.1 (9.9, 10.2)	11.1 (10.9, 11.2)	10.9 (10.4, 11.4)	3.3 (3.2, 3.5)
GWG > recommended	10.9 (10.7, 11.1)	10.7 (10.5, 10.9)	23.6 (23.0, 24.2)	0.3 (0.1, 0.4)
Overweight or obese (BMI ≥ 25) or GWG > recommended	20.2 (20.0, 20.4)	21.1 (20.9, 21.3)	31.8 (31.3, 32.4)	3.6 (2.4, 3.8)

*All women: BMI models adjusted for GWG and GWG models adjusted for BMI; also adjusted for maternal age, parity, weight-for-gestational age, prepregnancy health problems, antenatal care provider, low-income-cut-off, educational attainment, province of residence, ethnicity, support, stress and history of depression. Parity/previous caesarean subgroups: adjusted for all previous covariates except parity.

## Discussion

The nationally representative nature of the MES allowed us to estimate PAFs of caesarean births associated with overweight and obese BMI and above recommended GWG. We found that one in five (20.2%) caesarean births was associated with above normal BMI or excess GWG. Overall, a similar proportion of caesareans births was associated with above normal BMI and excess GWG (10.1% and 10.9%, respectively), but these proportions varied substantially with parity and previous caesarean status. As expected, the incidence of caesarean births increased with increasing BMI and GWG, with a stronger association with unplanned caesarean births. Although causality cannot be inferred due to the observational nature of our data, it is noteworthy that in principle, if the caesarean births associated with BMI and GWG were eliminated, Canada’s caesarean rate among singleton term pregnancies could be reduced by up to a fifth, e.g. from 25.7% to 20.6%. There is no consensus on an optimal caesarean rate. However, a rate of 20.6% in singleton term pregnancies would bring the overall caesarean rate closer to the 5%-15% range suggested by WHO [25].

Few previous studies have calculated the PAF of caesareans due to maternal weight. Lu et al. attributed 11.6% of caesareans in Alabama in 1995-1999 to obesity (> 29.0 kg/m^2^) at the first prenatal visit, an increase from 3.9% in 1980-1984 [14]. A Utah study attributed 38.8% of caesareans in 2001 to overweight or obesity at the time of birth thus also taking into account GWG [15]. Our PAF for overweight and obesity combined (10.1%) was similar to Lu et al.’s value for obesity alone, suggesting that a lower fraction of caesareans was attributable to high maternal weight in Canada in 2005-2006 compared to Alabama in 1995-1999. This is likely in part due to a lower Canadian prevalence of obesity (13.3%) than in Alabama (36.4%). Comparing our results to those in Utah is complicated by their use of maternal weight at birth as the determinant rather than BMI and recommended GWG. This methodological difference along with possible differences in the maternal weight distribution and obstetric practice in Utah and Canada may explain the much higher PAF observed in that study.

It is also noteworthy that compared to overweight and obese BMI, a similar proportion of caesarean births was associated with excess GWG due to the high prevalence of above recommended GWG. The additional risk posed by GWG was attenuated among parous women with previous caesarean births at high risk of repeat caesarean births, while it was magnified among parous women without a previous caesarean birth at low risk of caesareans. Among parous women without a prior caesarean birth the overall rate of caesarean birth was less than half the population rate; however, twice as many caesarean births were associated with above recommended GWG compared to overweight or obese BMI (23.6% versus 10.9%). Unfortunately, few women report being counselled about GWG [26]. This represents a missed opportunity for prevention, since health care providers are likely more able to impact GWG than BMI, as few women seek preconceptional care but most receive prenatal care within the first trimester [27].

Our study has some limitations. Although self-reported data on BMI and GWG are highly correlated with measured values, they tend to underestimate these values [28,29]. This could have resulted in overestimated associations between BMI, GWG and caesarean births [28]. Additionally, some residual confounding likely remains as we were unable to consider unmeasured factors. Data on indications for caesarean births would have increased our understanding of studied associations [30]. We were also not able to adjust for weight-related clinical conditions such as pre-eclampsia and diabetes, though there is some uncertainty about the degree to which such conditions are on the causal pathway, and should therefore not be adjusted for [4]. In addition, we made multiple comparisons which increases the chance of significant findings [31]; however, the associations noted in the results are plausible and we reported precise confidence intervals to support interpretation.

## Conclusions

In summary, our study found that one in five caesarean births in singleton term pregnancies in women 18 years and older was associated with above normal BMI or excess GWG, and this proportion is likely to increase as the prevalence of overweight and obesity rises. Nulliparous women with above normal BMI and excess GWG are at particular risk for unplanned caesareans. Strategies to reduce caesarean births in Canada must include measures to prevent overweight and obese BMI prior to conception and promote recommended weight gain throughout pregnancy.

## Competing interests

The authors declare that they have no competing interests.

## Authors’ contributions

SD and SD Mc conceived and guided the study. SD, J and RSK developed statistical methods and SD carried out statistical analysis. SD and SD Mc drafted and revised the manuscript on the basis of comments from other authors. All authors contributed to the interpretation of the data, critically reviewed all drafts of the manuscript and approved the final version submitted for publication.

## Pre-publication history

The pre-publication history for this paper can be accessed here:

http://www.biomedcentral.com/1471-2393/14/106/prepub
